# Comparing GPs’ risk attitudes for their own health and for their patients’ : a troubling discrepancy?

**DOI:** 10.1186/s12913-018-3044-7

**Published:** 2018-04-12

**Authors:** Antoine Nebout, Marie Cavillon, Bruno Ventelou

**Affiliations:** 1 ALISS, UR1303, INRA, Ivry-Sur-Seine, 94205 France; 2 KStat-consulting, Paris, 75014 France; 3 Aix Marseille University, CNRS, EHESS, Centrale Marseille, Aix Marseille School of Economics, Marseille, 13000 France

**Keywords:** Risk attitudes, GP’s behavior, Patient-regarding preferences, Representative sample, Medical decision making

## Abstract

**Background:**

In this paper, we report the results of risk attitudes elicitation of a French general practitioners national representative sample (N=1568).

**Methods:**

Willingness to take risks in four different domains (daily life, financial matters, own health and patient health) was collected through a large-scale telephone interview of GPs using self-reported 11-point Likert scale questions.

**Results:**

We uncover some specificities of the GPs population regarding their attitudes towards risk. In particular, we detect an important positive gap between their willingness to take risks in the domain of their own health and in the domain of the heath of their patients. This “patient-regarding” risk aversion is discussed with respect to its important consequences regarding medical behavior bias.

**Conclusions:**

We confirm the self-other discrepancy found in the medical literature on physicians’ behaviors and emphasize the utility of the study and measures of personality traits such as “risk attitudes” for the medical professions and for the population they address.

## Background

Understanding physician behavior and preferences is a central concern in health economics and psychology research. Indeed, their intrinsic preferences may have substantial consequences for their professional practices and decisions. There is a body of evidence in behavioral economics on physicians’ behavior such as their response to different incentives from payment schemes [1] or their patient-regarding motivation [2]. An important result of these studies is that the medical population (students or professionals) has unique characteristics with respect to both their professional motivation and their individual preferences.

In this paper, we focus on primary caregivers – specifically on general practitioners (GPs)– and reveal their preferences towards risk. The study of individual risk attitudes is an important field of research in behavioral economics [3] and has proven useful for explaining real-life behaviors [4, 5]. It is especially relevant for primary caregivers, as this population makes important decisions under risk on a daily basis (e.g., choices regarding curative treatments, further medical tests, hospitalisation, etc.). GPs’ medical behaviors might be determined to some extent by this psychological trait^1^. GPs’ willingness to take risks were elicited using self-reported Likert scale questions in different contexts, copying the scales validated in the German socio-economic panel [6]. In addition to the following three standard contexts (risk attitudes regarding their daily life, financial matters and their own health), we introduced a new context, i.e., risk attitudes regarding their patients’ health. Our motivation was twofold. First, most of the risky decisions made by GPs concern the health of their patients. Risk attitudes in this context are thus a natural candidate for explaining GPs’ medical behavior. Second, we sought to compare GPs’ risk attitudes regarding their own health and regarding the health of their patients.

If a gap between these two measures is found, this could raise some interesting questions regarding the efficiency of medical services because a patient might expect his GP to address his situation with the same level of willingness to take risks that the GP would with his own [7–9]. There is however an extensive body of studies on medical decisions for selves vs. others showing that choices may differ depending on who is the object of the decision and what is the decision maker role [10]. For example, US primary care physicians tend to recommend treatments with greater chance of survival (and of complications) to patients more often than they chose it for themselves [11].

For what concerns willingness to take risks for selves versus others, most of the existing studies relates to financial issues [12, 13], relationship [14] or physical safety domains [15].

We extend this line of research taking into account that risk taking behaviors are domain-specific and dependent on the targeted population [16], so the existing results in the monetary domain may not apply to the health domain and results on the general population to the GPs’. In this paper, we report the results of a representative national panel survey on the risk attitudes of 1568 French GPs in four different contexts using the same 11-point scale than [17]. We measure the association between socio-demographic variables such as gender, age, location and volume of activity and the measured risk attitudes. These analyses allow us to present original results on the specificity of the physician population with respect to attitudes towards risk. Finally, the within-subjects comparison of our results between the “patient health” and the “physician’s health” contexts suggests that GPs are significantly willing to take more risk in situations affecting their own health than in those affecting the health of their patients. In fact, following the shared decision making paradigm, the GP should make a recommendation that matches his patients’ preferences [18]. Consequently, if the discrepancy between GPs’ risk attitudes for their own health and for their patients’ matches a real difference of risk attitudes between the GP and the general population, there is not issue in terms of public health. But, the discrepancy between GPs’ own and others risk attitudes may also be inadequate and due to a systematic underestimation of the willingness to take risks of their patients by the health professionals. In this case, this quasi-systematic difference is worth exploring because it might have the effect of extensively biasing the terms of the medical decisions and recommendations.

Our results are not in line with the existing psychological literature in the monetary domain [12]. We therefore discuss and interpret the specificity of our result in the health domain and find that consistency with the extant literature on self-other differences in medicine [19, 20]. The remainder of the paper proceeds as follows. “Methods” section presents the sampling procedure for the panel and the questions used to measure risk attitudes. “Results” section presents our results, which are discussed in “Discussion”section.

## Methods

### Sampling

In 2008, approximately 58 000 GPs (31.6% of whom were women) were in private practice in France. The survey described in this paper was the fifth and last in a series nested in the national panel of French GPs, which was designed to collect data regularly on their activities and practices.

Composed of a national sample and three regional oversamples (Burgundy, Pays de la Loire region and Provence-Alpes-Cote d’Azur), the French GP panel was constituted in June 2010 through a partnership involving the research department of the Ministry of Health, the health observatories and the representatives of self-employed GPs of the three regions mentioned above. The sampling frame was obtained from the Ministry of Health’s exhaustive database on health professionals in France. Matching this survey with data from the General Health Insurance Fund made it possible to retain only the GPs who received a fee of at least one euro during the year. Physicians planning to cease their activities or move within one year and those with a full-time special mode of practice (acupuncture, homeopathy, etc.) were excluded from the sampling frame.

Sampling was stratified for location of the general practice (urban, peri-urban, or rural areas), gender, age (<49 [Q1], 49-56, >56 years old [Q3]) and annual volume of activity, defined by the number of consultations^2^ (<2849 [Q1], 2849-5494, >5494 [Q3])^3^ in 2008. Information on each GP was obtained from the General Health Insurance Fund.

To limit selection bias that might have resulted from particular opinions/attitudes, the specific topics to be studied were not mentioned to the GPs before they were asked to participate in the panel.

The fifth wave of the survey took place during the first trimester of 2013. In total, 2077 physicians were contacted by mail and then by telephone. Professional investigators interviewed the panel members with computer-assisted telephone interview (CATI) software.

### Ethics statement

GPs who agreed to participate in the panel returned signed, written consent to our team. The National Data Protection Authority (Commission Nationale Informatique et Libertes), responsible for ethical issues and the protection of individual data in France, approved the panel and its procedures.

### Procedure and questionnaire

**Fig. 1:**
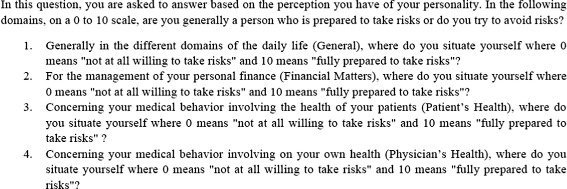
Likert questions used to measure GP’s willingness to take risks

Risk attitudes were measured by using four self-reported Likert scale questions inspired by [17]. We elicited GPs’ willingness to take risks in four different contexts (regarding their daily life, regarding financial matters, regarding their own health and regarding the health of their patients) on an 11-point scale. Figure 1 presents a literal translation of the questions asked by the interviewers.

For each context, GPs were allowed to select a “Do not know” option if they were unable or reluctant to respond to the question. Note that the wording of the questions was identical to those of [17] with the only difference^4^ being that these questions were asked via telephone, meaning that the interviewee did not have to tick a box on a 0-10 scale but instead to report a figure between 0 and 10 to the interviewer. However this methodological difference should not be too dramatic since, as far as risk attitudes are concerned, telephonical interviews proved to be as efficient as face-to-face interviews [21].

The order of the questions for the first two contexts (general and then financial matters) was identical across the entire sample, whereas half of the sample was interrogated on the patient’s health domain first and the other half on the physician’s health domain first.

## Results

**Fig. 2:**
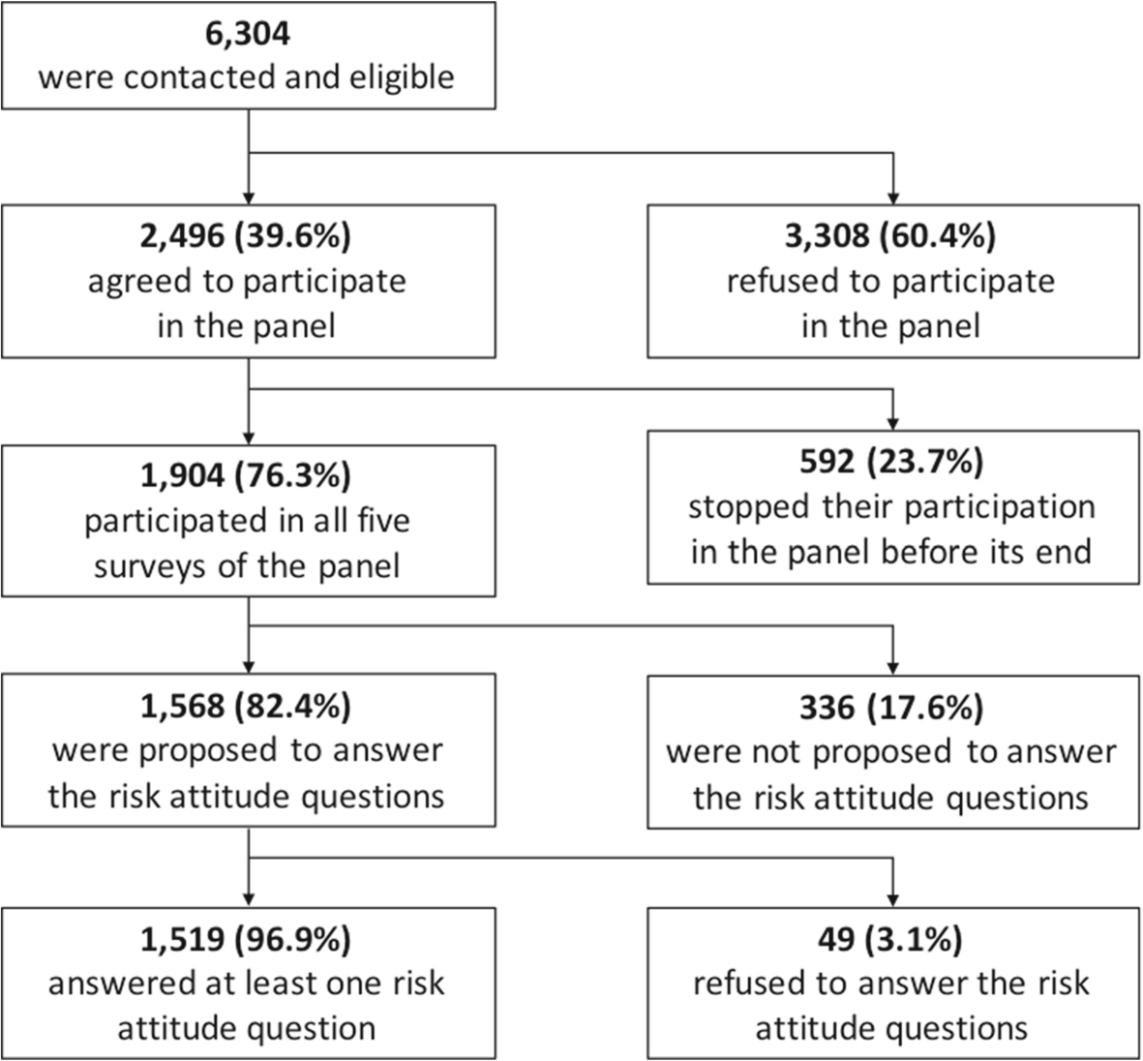
Respondents’ sample description

Ultimately, 1904 out of 2077 GPs (92% response rate) responded to the survey. The analyses presented in this study are based on the national sample (1052 respondents) and the two, Burgundy (201) and Provence-Alpes-Cote d’Azur (315), oversamples for a total of 1568 respondents^5^. Sample selection is described in more details in Fig. 2.

Descriptive results of the risk attitudes for the four different contexts are presented in Table 1. Among the 1568 surveyed GPs and for each context, a rather limited share (between 3 and 4%) chose the “Do not know” option. The two contexts for which GPs are the least willing to take risks are financial matters and patient’s health. Risk attitudes are not perfectly correlated across contexts, but the pairwise correlations are large and are all highly significant (Table 1). This suggests a stable underlying risk trait that is nevertheless sensitive to the context. In particular, it means that physicians who are generally risk averse are also more risk averse regarding their patients^6^.

**Table 1:** Means and correlations (Kendall’s coefficient) of GP’s risk attitudes in different contexts

	General	Financial matters	Patient’s health	Physician’s health
Mean	4.78	3.78	3.31	5.13
*(Standard Deviation)*	*(2.28)*	*(2.36)*	*(2.29)*	*(2.41)*
Mean (Men)	4.92	3.88	3.42	5.15
Mean (Women)	4.43	3.51	3.02	5.10
General	1.000			
Financial Matters	0.441^c^	1.000		
Patient’s Health	0.378^c^	0.368^c^	1.000	
Physician’s Health	0.338^c^	0.293^c^	0.335^c^	1.000
“Do not know”	49 *(3.12%)*	61 *(3.89%)*	59 *(3.76%)*	55 *(3.51%)*
Observations (N)	1519	1507	1509	1513

^c^, ^b^ and ^a^ represents significance at a 1‰, 1% and 5% level respectively

In Table 2, we investigate the possible socio-demographic determinants of GPs’ individual risk attitudes and we present the results of the four OLS regressions of risk attitudes in each context on the four variables used for stratification: gender, age, volume of activity and location.

**Table 2:** OLS regressions of the willingness to take risks on socio-economics variables

	Willingness to take risks			
Variables	General	Financial matters	Patient’s health	Physician’s health
*GP’s Characteristics*				
Constant	4.602^c^	3.670^c^	3.189^c^	4.470^c^
	*(0.204)*	*(0.212)*	*(0.206)*	*(0.215)*
Gender				
Male	*Ref*.	*Ref*.	*Ref*.	*Ref*.
Female	-0.345^b^	-0.330^b^	-0.252^a^	0.105
	*(0.139)*	(0.144)	(0.140)	(0.147)
Age				
<49	*Ref*.	*Ref*.	*Ref*.	*Ref*.
49-56	0.189	-0.083	0.035	-0.008
	*(0.140)*	*(0.145)*	*(0.141)*	*(0.148)*
>56	0.368^b^	-0.067	0.401^a^	0.175
	*(0.156)*	*(0.162)*	*(0.157)*	*(0.165)*
Volume of activity				
<2849	*Ref*.	*Ref*.	*Ref*.	*Ref*.
2849-5494	0.064	0.117	0.179	0.350^b^
	*(0.150)*	*(0.156)*	*(0.152)*	*(0.159)*
>5494	0.274	0.370^b^	0.332^a^	0.722^c^
	*(0.173)*	*(0.180)*	*(0.174)*	*(0.183)*
Location				
Rural	*Ref*.	*Ref*.	*Ref*.	*Ref*.
Peri-urban	-0.044	-0.040	-0.198	0.349^a^
	*(0.180)*	*(0.187)*	*(0.182)*	*(0.191)*
Urban	0.006	0.163	-0.135	0.246
	*(0.146)*	*(0.152)*	*(0.148)*	*(0.155)*
Observations (N)	1519	1507	1509	1513

^c^, ^b^ and ^a^ represents significance at a 1‰, 1% and 5% level respectively

Except in the physician’s health domain where no significant effect is found, women are stating significantly less willingness to take risks than men. In the general and patient’s health domains, the oldest GPs report a significantly greatest willingness to take risks than the younger GPs^7^. The volume of activity is also a relevant variable since the most active GPs declare significantly more willingness to take risks in all the domains except the general one.^8^ Finally, the location of exercice is never a significant explanatory variables of the individual GPs’ propension to take risks (except for peri-urban GPs who declare more willingness to take risks in their own health domain).

## Discussion

Through a large-scale telephone interview, we elicited risk attitude metrics for each doctor using direct stated preferences to take risks in four different contexts. We measured the patient-regarding preferences of GPs by asking similar questions involving their own health and the health of their patients.

Using this specific population of GPs, we checked and verified results that were already found in the literature. However, we also obtain results that demonstrate the particularities of the physisian population. We comment in more details these results with regard to gender, age and health domains.

### Gender effect

Gender difference in risk attitudes have been the object of numerous empirical findings in behavioral economics (for literature reviews, see [22, 23]) and psychology [24]. In most of these studies, women are found to be more risk averse than men [25]. This result holds when using Domain-Specific Risk-Taking Scale, DOSPERT [26] or self-assessed Likert measures of risk attitudes in cross-sectional surveys [17]. For GPs, we observe significantly more risk aversion among women in the general and financial^9^ contexts and extend this result to the patient’s health domain. However, we do not observe such a gender effect in the physician’s health domain, unlike in [17] where women are more risk averse than men concerning their own health. This confirms that the personal health domain seems to be a context in which the GPs substantially depart from the general population with respect to risk attitudes. Such differences with the general population are also observed for specific populations when risk attitudes are measured in their domains of expertise or practice [16].

### Age effect

There are several studies in behavioral economics [17, 27] revealing a gradually lower willingness to take risks across the life span in cohorts, suggesting that individuals become more risk-averse as they grow older. Recent cross-cultural psychological meta-study [28] confirms this empirical result that appears to be robust to the risk attitude elicitation method [26, 29].

In our GPs’ panel we oberve a significant age effect in the general and patient’s health domains. Interestingly, in these two domains, older GPs are less risk averse than are younger ones. No significant effect of age is found in the financial and GP’s own health domains. Regarding the general domain, this result contradicts the common finding in the literature and highlights the particularities of the GP population. For example, the hypothesis proposed by [30] or [31], which explains this age effect on risk attitudes by a decrease in cognitive abilities due to aging, may not apply to our surveyed population of GPs since only 5% of the interrogated GPs are over 70. It should also be noted that the mean age in our sample is 50.1 years, std 9.6, min 29, max 76 which is a rather limited age range. In addition, all the interrogated GPs are active, even the older ones (who also have the higher volume of activity and revenue).

Concerning the effect found in the patient’s health domain, two interpretations are possible: on the one hand, the older physicians may be willing to take more risks for their patients because they are more experienced and potentially aware of the upsides of, occasionally, pursuing risky options. On the other hand, a generational effect could explain this result. Older GPs may be less concerned by possible lawsuits in the event of medical errors or may have an extremely self-centered approach to the doctor-patient relationship due to their antiquated medical education, which induces a greater willingness to take risks involving their patients.

It is however important to note that whatever age category is considered, GPs take significantly more risk regarding their health than regarding the health of their patients; this point is addressed in greater detail in the next section.

### The within discrepancy among domains of health

A troubling result of the study is that GPs’ willingness to take risks involving their own health appears to be much higher than for the general population and appears much higher than the risk they declare to be willing to take for their patients, creating a discrepancy between self and others. In addition, unlike in [17], we do not find any gender nor age effect in this domain. The particularity of the GP population is, thus, really striking.

The domain in which GPs are the most reluctant to take risks is their patient’s health^10^. The discrepancy between the GPs’ risk attitudes regarding their own health^11^ and that of their patients is extremely large and might have significant consequences for medical behaviors. Indeed, we can legitimately imagine that a GP would not have the same prescriptive behavior for himself as for his patient when facing the same medical symptoms, although patients often expect their physician to make the same decision for them as he would for himself. This result showing that GPs are more risk averse when they are addressing the health of their patients could be interpreted in different ways:

1. *An economic rational* A first explanation of this result could be that physicians take less risk when the health of their patient is involved because this type of risk taking might lead to lawsuits and potential monetary losses. Given the French medical system, in which such lawsuits are extremely rare, this interpretation can be excluded. However, we conjecture that the gap we found is likely to be more pronounced in countries where the legal system is harsh towards medical caregivers.
2. *A social desirability bias* During interviews, doctors intentionally may have reported a discrepancy between patient’s health and own health risk attitudes because they believed they “politically” had to. In our view, this interpretation can be eliminated because the order of questions regarding “own health” and “patient’s health” was randomized. Because we find no order effect^12^, the measured gap cannot be a consequence of “desirable” response behavior^13^ intended to create a contrast (when GPs respond to the first question, they do not know that they will have to respond to the second one)
3. *A paternalistic bias* In their daily practice -and not only in interviews- doctors do not act for their patients as they would act for themselves. In our framework, this paternalistic attitude is not what [32] call “asymmetric” or “libertarian paternalism”, which would be desirable for the patient. In fact, our finding may imply a real decline in opportunities and medical options proposed to patients. Indeed, GPs explicitly recognize that they support an higher degree of risk for themselves that they would suggest their patient to take, potentially reducing the scope of medical options they will advise them to consider. That is why we would rather qualify this discrepancy between own’s and patient’s risk attitudes of “paternalistic biais” rather than libertarian paternalism.
4. *A self-correction of the pre-existing gap* The fourth interpretation assumes that GPs substantially differ from the general population with respect to risk attitudes in the health domain (GPs are more willing to take risks). Thus, an (highly optimistic) interpretation could be that GPs, reporting safer attitudes for patients than for themselves, tend to rectify the gap that they know there exists between them and the general population. This gap could have several origins: GPs differ from patients in their medical knowledge and information^14^, as well as in their access to care. Thus a physician may take greater risk for his health, as he is certain to receive appropriate assistance in the event of an adverse outcome^15^. Aware of this gap, the GP would then be attempting to act as a “perfect agent” of the patient [33] and establish the proper attitude using a sophisticated adjustment. In this situation, the GP would dodge any preferences’ diagnosis and the gap highlighted would have adverse consequences for the healthcare system. Indeed, following the paradigm of health professionals based on patient-centered communication and shared decision-making, the physician should not make the decision based on what seems as the proper decision for himself but based on what seems right for the patient.

Naturally, this last interpretation would require the investigation of risk attitudes in a representative sample of the French population to confirm the existence of the GP/patient risk attitude gap in the health domain^16^.

Another limitation of our study is the type of metric we used to measure risk attitudes, i.e., a self-assessed measure of willingness to take risks on a 0-10 scale. These survey measures are, by construction, not incentivized, which could be an important concern regarding the measurement of risk attitudes in the monetary domain. However, [17] showed that, in a survey-experiment using a sub-sample of their population, their general risk question was significantly correlated with the measure derived from incentivized binary lottery choices, suggesting that these two methods indeed measured the same psychological trait. We therefore assume that this relationship holds in our survey and allows for a meaningful interpretation of our results in terms of risk attitudes.

Concerning the health domains, incentivization of the questions is impossible both in survey measures and in “quantitative” measures of risk attitudes involving lottery choices with consequences framed in terms of health. In a companion paper, [34] elicited GPs risk attitudes using hypothetical binary lottery choice questions with three different attributes (money, own health and patient’s health). In the two health domains, GPs had several binary choices to make between two therapies: one safe therapy that provides a certain amount of additional years of living in good health and a risky therapy that provides an higher number of additional years of living in good health with probability *p* and nothing with probability 1−*p*. Using a between-subject analysis, [34] find a similar systematic discrepancy between the GPs’ willingness to take risks for their patients’ health and for their own. Although their measures are also based on hypothetical choices, they rely on experimental technics that are commonly used in behavioral and experimental economics for measuring risk attitudes in various domains [35]. It is thus reassuring to find the same qualitative result with both types of measures since this study is the first to collect attitudes in the patients’ health domain using a Likert scale measure of willingness to take risk^17^.

Finally, [36,37] use the risk attitudes measures presented in this paper as explanatory variables of actual medical behaviors. Michel-Lepage et al. [36] find that risk-averse GPs use more Rapid Antigen Diagnostic Tests (RADTs) in tonsillitis in children, and [37] find that risk-averse GPs were more often vaccinated against seasonal and pandemic influenza, more often recommended the pandemic influenza vaccination and were also more in favor of vaccination in general. Concerning the measure of willingness to take risks for the health of their patients (that has been introduced in this study for the first time), [38] find that it is associated with two medical practices (no prescription of antibiotics and update of a booklet) which suggests a good external validity of this question. The statistical significance of these risk attitude scales in the explanation of professional medical behaviors emphasizes the utility of the study and measures of this personality trait –risk attitudes– for the medical professions and for the population they address. Furthermore, the potential influences of GPs’ individual characteristics on clinical decision making [28], also confirmed by our study (through age, gender and activity effects on GPs’ risk attitudes), may lead to individual physician practices variation part of which are (probably) not desirable [39]. In highlighting the specificities of the GPs’ population (especially an unconventional age effect [26]), our study may help to understand and prevent potential medical practice variations among the French GPs’ population and to provide solutions for targeting “at risk” GPs with personalized practice recommandations.

Our main point concerns the self vs patients gap found in this study which extends the existing findings in self versus others medical decision making [10,11] and in other domains [12,14]. In fact, this gap could be detrimental when the gap is not justified by a difference of preferences between GPs and the general population. In this case, the GP tend to underestimate their patients’ willingness to take risks and medical conflicts that may arise between doctors and patients [40], as the core of the patients’ preferences i.e. true risk attitudes, would be neglected. The GPs may recommend them medical options that do not match their risk attitudes, involving a deep public health issue [41]. To account for the relevance of this issue, we must check the reality of the gap and evaluate clearly its precise magnitude; only a systematic study of the difference between actual patients’ risk preferences and GPs’ risk preferences for their patients would be able to provide this precise measurement (see for example [42], which used mirrored questions).

## Conclusions

This study is, to the best of our knowledge, the first cross-sectional representative survey that elicits behavioral characteristics of GPs in addition to socio-demographical and professional information. In this article, we focus on a specific psychological trait; *the willingness to take risks;* which is elicited via self-assessed likert scale questions in four domains: general, financial, own health and health of the patients. We highlighted some specificities of the GPs’ population (age and gender effects) and our main result shows a significant discrepancy between the GPs’ wilingness to take risks regarding their own health and that of their patients. Our data do not allow us to test if this discrepancy corresponds to a real difference between the GPs’ and the general population’s risk preferences towards health. If this is not the case and GPs risk attitudes towards their patients’ health do not match their patient’s preferences towards health, this could lead to systematic patients’ preferences misdiagnosis and therefore to healthcare provision inefficiency. Further research, simultaneously measuring risk attitudes of GPs and of their patients, are therefore required to determine the extent of the issue highlighted in this study.

## Abbreviations

CATI: Computer-assisted telephone interview
GP: General practitioner
RADT: Rapid antigen diagnostic test

## Footnotes

1 An example of field study on surgeons is presented in [43].

2 House calls and at the practice.

3 The cutting points were chosen by quartiles defined on the number of acts per year: First quartile-limit is 2849 acts per year, and defines the upper-bound for first quartile (GP with low level of activity). The third quartile is 5494 acts per year and defines the lower-bound for the fourth quartile (GP with high level of activity). The middle class is between 2849 and 5494 acts and defines the 50% of the GPs who have an “intermediary” level of activity.

4 We also add the “in the different domains of the daily life” in the general domain following requests from many interviewees of the pilot asking for more details on this specific question.

5 The remaining 336 GPs of the Pays de la Loire oversample were proposed another questionnaire with specific questions on regional issues.

6 This suggests that own perceptions of the appropriateness of risk taking also affects behavior towards patients.

7 This result holds using the continuous age variable in the regression instead of the stratification age variable described above.

8 The highest volume of activity (>5494) has a significant positive impact on the willingness to take risks in the financial domain. This could be explained by the fact that GPs with higher activity are wealthier and consequently take more risks [17].

9 Note that, in 2004, french women GPs were earning 33% less than men GPs [44].

10 This result is contradictory with studies showing that some GPs may be, in practice, take too many risks on behalf of the patients which can lead to detrimental consequences for the patients [45, 46].

11 Such risk seeking behavior of GPs concerning their own health is consistent with studies showing that physicians are reluctant to seek help for their own medical issues [47, 48].

12 For patient’s health, *p*=0.18 for the unpaired t-test between the two conditions, *p*=0.29 for physicians’ health and *p*=0.84 for the difference between patients’ and physicians’ health.

13 We recognize that GPs’ way of responding to the questions may be a reflection of an internalized narrative effect regarding the asymmetrical patient-physician-relationship and the identity of a physician [49]. Only a mirror study with cross collection of data, both on patient’s preferences and on the GPs beliefs of the patient’s preferences could clarify this point.

14 And eventually in their ability to correctly threat and interpret this information (although [18] emphasize that doctors may also fall into systematic biases in their statistical analysis).

15 GPs might also have better cognitive abilities than the general population, thus explaining the gap because, as suggested by a referee, cognitive performance and risk attitudes may be related [50].

16 As suggested by a referee, a test for differential item functioning would be relevant for the likert scale question assessing willingness to take risks in the health domain as it cannot be excluded that this item functions differently in the general population and in GPs.

17 Actually, the reader has to accept the idea that a complex issue such as willingness to take a risk on behalf of a patient can be measured by one question phrased in general terms whereas patients’ recommandation behavior relies on the GP’s self-awareness concerning a complex pattern of motives. This is anyway in the line of the results of Dohmen *et al* (19), who coined the principle that a simple scale elicitation tool could be as efficient as a complex tool, when collecting attitudes toward risk.
